# Larval Behaviours and Their Contribution to the Distribution of the Intertidal Coral Reef Sponge *Carteriospongia foliascens*

**DOI:** 10.1371/journal.pone.0098181

**Published:** 2014-05-22

**Authors:** Muhammad Azmi Abdul Wahab, Rocky de Nys, Nicole Webster, Steve Whalan

**Affiliations:** 1 AIMS@JCU, James Cook University, Townsville, Queensland, Australia; 2 School of Marine and Tropical Biology, James Cook University, Townsville, Queensland, Australia; 3 Australian Institute of Marine Science, Townsville, Queensland, Australia; 4 Marine Ecology Research Centre, School of Environment, Science and Engineering, Southern Cross University, Lismore, NSW, Australia; Victoria University Wellington, New Zealand

## Abstract

Sponges (Phylum Porifera) are an evolutionary and ecologically significant group; however information on processes influencing sponge population distributions is surprisingly limited. *Carteriospongia foliascens* is a common Indo-Pacific sponge, which has been reported from the intertidal to the mesophotic. Interestingly, the distribution of *C. foliascens* at inshore reefs of the Great Barrier Reef is restricted to the intertidal with no individuals evident in adjacent subtidal habitats. The abundance of *C. foliascens* and substrate availability was first quantified to investigate the influence of substrate limitation on adult distribution. Pre-settlement processes of larval spawning, swimming speeds, phototaxis, vertical migration, and settlement to intertidal and subtidal substrate cues were also quantified. Notably, suitable settlement substrate (coral rubble) was not limiting in subtidal habitats. *C. foliascens* released up to 765 brooded larvae sponge^−1^ day^−1^ during the day, with larvae (80%±5.77) being negatively phototactic and migrating to the bottom within 40 minutes from release. Subsequently, larvae (up to 58.67%±2.91) migrated to the surface after the loss of the daylight cue (nightfall), and after 34 h post-release >98.67% (±0.67) of larvae had adopted a benthic habit regardless of light conditions. Intertidal and subtidal biofilms initiated similar settlement responses, inducing faster (as early 6 h post-release) and more successful metamorphosis (>60%) than unconditioned surfaces. *C. foliascens* has a high larval supply and larval behaviours that support recruitment to the subtidal. The absence of *C. foliascens* in subtidal habitats at inshore reefs is therefore proposed to be a potential consequence of post-settlement mortalities.

## Introduction

The effective management and conservation of marine species requires a holistic understanding of the physical, biological and ecological processes influencing populations [1]–[3]. Sponges (Phylum Porifera) are abundant, diverse and play important functional roles in many aquatic ecosystems [4], [5]. However, despite their evolutionary and ecological significance, sponge population demographics are relatively understudied [6], [7]. For sessile marine invertebrates such as sponges, the mobile larval phase is critical to population maintenance and biogeography [2], [8], [9].

Dispersal over large geographic ranges is unusual for sponges (but see [10] for mechanisms facilitating large scale dispersal), with most sponge larvae exhibiting short competency periods (minutes to <2 weeks) and restricted dispersal potential [11], [12]. Due to the restricted swimming capabilities of sponge larvae [13]–[16], hydrodynamic processes are likely to play an important role in larval dispersal [17], [18]. Nevertheless, innate larval behaviours in response to environmental factors can indirectly contribute to horizontal larval dispersal. For example, phototaxis (attraction to light) can position larvae in bodies of water of differing flow regimes through light-directed vertical migration [15]–[17], [19]–[23].

Given these properties, the adult distribution of sessile invertebrates can be strongly influenced by larval settlement and post-settlement processes. However, larval settlement also relies on the availability of suitable substrate for attachment and metamorphosis [3]. For sponges, the availability of solid and stable substrates such as coral rubble is critical for primary attachment to the benthos [24]. Furthermore, the availability of substrate associated cues such as microbial biofilms, induce faster and more successful metamorphosis in sponge larvae [15], [16], [25]. Finally, post-settlement pressures such as predation [26], [27], competition [28], [29], food availability [30], [31] and light quality (for species possessing phototrophic symbionts, [32], [33]) also influence adult population distributions and demographics.

*Carteriospongia foliascens* is an abundant, conspicuous and widely distributed Indo - Pacific phototrophic sponge, and is found across a range of intertidal and mesophotic habitats of the Great Barrier Reef (GBR) ([34]–[36], see [37] for conspecificity between intertidal populations, studied herein, and subtidal populations). *C. foliascens* is a brooding species and although reproductive all year round exhibits peak reproduction during the Austral summer from October to December [38]. Within the Palm Island region of the central GBR, *C. foliascens* occurs solely on intertidal reef flats and is absent from adjoining sub-tidal reefs (this study). To understand the potential of larval behaviours in contributing to adult distributions, this study firstly investigated patterns of larval spawning, swimming speeds, phototaxis and vertical migration. Secondly, the distribution of adult sponges and substrate availability was measured across habitats. Thirdly, the effects of biofilms (i.e. intertidal and subtidal) on larval settlement and metamorphosis were quantified.

## Materials and Methods

### Study sites and benthic surveys

All field surveys and collections were conducted under the Great Barrier Reef Marine Park Authority Permit #G12/35236.1 and did not involve any endangered or protected species. The distribution and abundance of adult *Carteriospongia foliascens*, was determined on the reef flats of Little Pioneer Bay, Orpheus Island (18°36.989′S, 146°29.832′E) and north Juno Bay, Fantome Island (18°41.405′S, 146°31.272′E), central Great Barrier Reef (GBR). Surveys were undertaken in January and March 2012 using belt transects (500 m×2 m, n = 3) oriented from the shoreline edge of mangroves (shallow, intertidal) to the reef slope (deep, subtidal). Positions of sponges along transects were recorded. Water depth (cm) was recorded every 10 m along transects and adjusted relative to the tidal datum by subtracting *in situ* water level from predicted tide levels (National Tide Centre, Australian Bureau of Meteorology; http://www.bom.gov.au).

Adult *C. foliascens* are commonly found attached to coral rubble. To identify whether substrate availability was contributing to sponge distributions, additional surveys for substrate type (sand, coral rubble and live coral) were conducted at Little Pioneer Bay (Orpheus Island), Hazard Bay (Orpheus Island, 18°38.417′S, 146°29.789′E), north Juno Bay (Fantome Island) and south Juno Bay (Fantome Island, 18°41.130′S, 146°30.880′E). Belt transects (25 m×2 m, n = 3) were oriented along three depth profiles (+100 cm, +50 cm and −100 cm) from the tidal datum (FTD). Depth profiles were selected based on the previous survey, which identified mean depths where sponges naturally occurred (+50 cm) and depths where sponges were absent (+100 cm and −100 cm). A 1 m×1 m quadrat, divided into a 4×4 grid layout, was used to systematically quantify substrate composition at 5 m intervals along transects. Area estimations for substrate type were conducted by the same observer to maintain consistency. Additionally, to quantify the realized available space for larval settlement, the proportion of coral rubble accessible for settlement (i.e. free of other macro-invertebrates and macroalgae) was recorded at +50 cm FTD at north Juno Bay and Little Pioneer Bay in January 2012.

### Larval release and collection

*C. foliascens* is a brooding sponge that spawns tufted parenchymella larvae. Although this species is reproductive throughout the year, increased reproduction occurs during the Austral summer months from October to December [38]. Fourteen reproductive sponges were collected from Little Pioneer and south Juno Bay to provide larvae for experiments in October 2011. Sponges were maintained in aquaria receiving flow through 10 µm filtered seawater (FSW) at Orpheus Island Research Station (OIRS) until spawning. Larvae were collected from adult sponges using mesh traps following [16].

Preliminary investigations found no release of larvae at night between 1800 h and 0600 h. To quantify larval release, larval traps were placed over fourteen sponges maintained in flow-through aquaria at OIRS at 0600 h. Larvae were subsequently collected and counted at three hour intervals (0900 h, 1200 h, 1500 h and 1800 h) over seven days. Representative larval samples (n = 400) were preserved in 2.5% glutaraldehyde (in FSW) for size measurements.

### Larval swimming ability

Larval swimming speeds were assessed at 0, 2, 4, 6, 12, 18 and 24 h post-release. A glass aquarium (50 cm×5 cm×5 cm) superimposed with a 1 cm×1 cm grid on the bottom, filled with artificial seawater (ASW, 35 ppt, Tropic Marin sea salt in distilled water) was used as a swim chamber. Larvae (n = 10) were videotaped using a Sony video camera (DCR-DVD101E) at each time period for 1 min and subsequently removed from the experiment. Larval speed (cm.s^−1^) was determined by tracking larval horizontal swimming distance over time using the plugin MTrackJ in ImageJ 1.46r (National Institutes of Health, USA).

### Pre-settlement behaviour

#### Behaviour of “newly released” larvae

“Newly released” larvae refer to larvae released within 1 h from adult sponges at the start of experiments. To investigate larval vertical migration behaviour in response to light (phototaxis), 1000 ml graduated cylinders (height 46 cm and diameter 6.5 cm) filled with 1000 ml of artificial seawater were used as experimental columns. Artificial seawater (ASW) was used for all experiments to exclude influences of waterborne cues on larvae. Natural daylight was used as a light source for all phototaxis experiments. Light intensities ranged between 0.2 kW m^−2^ and 276.9 kW m^−2^ between 0600 and 1800 h daily (HOBO Pendant UA-002-64, One Temp Pty Ltd, Massachusetts).

Each experimental column was divided into two, one half covered in foil to exclude daylight and the other half uncovered for light penetration. Light treatments consisted of natural daylight presented to 1) the top half of the experimental column (n = 3) and 2) the bottom half of the column (n = 3) following [16]. Twenty larvae were introduced to the surface of each experimental column using a pipette and columns gently swirled to disperse larvae throughout the water to remove any bias of initial static water conditions and larval placement. The columns were then positioned in flow - through water baths to maintain ambient water temperature (≈25°C). The number of larvae in the light exposed half of each column was recorded at 0 (5 mins post-swirling), 20, 40, 60, 80, 100, 120, 140, 160, 180, 200, 220 and 240 min.

#### Behaviour of 4 h old larvae

To investigate changes in vertical migration behaviours in older larvae, the effect of partial light exposure to the top (n = 3) and bottom (n = 3) portion of each column was assessed on larvae older than 4 h (n = 50) following methods described previously. The number of larvae in the light exposed half of each column was recorded at 0, 2, 4, 6, 12, 18, 24, 30, 36, 42 and 48 h.

To examine larval vertical migration behaviour in the dark, a second experiment was designed to remove phototactic cues entirely. Experimental columns and number of larvae used (n = 50) were consistent with experiments already described. For dark treatments, daylight was excluded from experimental columns (n = 3) by covering columns entirely with aluminium foil. For light treatments, experimental columns were exposed to full daylight (n = 3). Positions of larvae in the top and bottom half of each column were recorded at 0, 2, 4, 6, 12, 18, 24, 30, 36, 42 and 48 h. Larval positions were assessed in a dark room using a red-filtered torch for the dark treatment and at night for the light treatment. Any effect of light during sampling on larval behaviour (for dark treatment and at night) was assumed to be negligible due to the low intensity and short exposure.

### Settlement behaviour

Settlement and engagement in metamorphosis were assessed independently in all experiments. Settlement refers to a larva that has attached itself to the substrate via its anterior end and which was not dislodged with gentle agitation of the experimental chamber (e.g. swirling). Engagement in metamorphosis is defined as a distinct change in larval morphology through flattening of the posterior half to assume a hemispherical form (see Video S1 for a time-lapse video clip of *C. foliascens* engagement in metamorphosis). For ease of communication, engagement in metamorphosis is referred to as metamorphosis hereafter.

#### Gregariousness

The response of larvae to conspecific cues (both adult and larval derived) was tested prior to the main settlement assays. The potential effect of adult waterborne conspecific cues was first assessed by exposing larvae to 1) adult infused ASW (ai-ASW) (n = 12) and 2) sterile ASW (control; n = 12). Adult infused ASW was prepared by maintaining three palm-sized sponges in five litres of ASW without water exchange for eight hours. Aeration was supplied to avoid anoxic conditions. Sterile polypropylene jars (Sarstedt – 70 ml) were employed as experimental chambers and 40 ml of experimental ASW was used in each replicate. Larvae (up to 4 h old; n = 10) were introduced to each jar using a pipette. A 1 cm diameter hole in the lid of each jar provided gas exchange. Jars were placed into outdoor water baths to maintain natural daylight and photoperiod, and ambient water temperature. Larvae that were active, settled, metamorphosed or dead were assessed at 0, 1, 3, 5, 7, 10, 15, 25, 35 and 45 h.

To elucidate effects of larval settlement in the presence of conspecifics, a second experiment was designed to test the influence of larval density on settlement and metamorphosis. Treated, sterile, polystyrene 6-well plates (Iwaki) filled with 10 ml of ASW were used as experimental chambers. Densities of 1, 2, 5, 10 and 20 larvae well^-1^ were selected following [16]. To facilitate frequency analysis, the total number of larvae (4 h old; n = 100) used in each density treatment was kept consistent thus allowing for comparisons of solitary and multiple larvae on settlement and metamorphosis (therefore 100 wells for 1 larva well^−1^, 50 wells for 2 larvae well^−1^, etc.). Experimental chambers were maintained in an outdoor water bath and larval condition recorded at 0, 1, 3, 5, 7, 10, 15, 20, 25, 30, 35 and 40 h.

#### Effects of biofilm origin on settlement and metamorphosis

To assess larval settlement and metamorphosis to intertidal and subtidal habitats, larvae were presented with surfaces conditioned with 1) intertidal biofilms (n = 10) and 2) subtidal biofilms (n = 10). Polypropylene jars (Sarstedt – 70 ml) were placed in reefal habitats for six weeks to allow sufficient time for biofilm development. Intertidal treatments were left on the intertidal reef flat of Little Pioneer Bay (where adult *C. foliascens* occur naturally) and subtidal treatments were established at 3 m depth on an adjacent reef slope, a habitat not known to support adult populations. Fouling macro-invertebrates and filamentous macroalgae growing within experimental jars after six weeks were carefully removed using a fine brush and pair of forceps prior to the experiment, to avoid confounding effects of fouling organisms on larval settlement. Sterile jars (n = 10) were used as controls. Forty millilitres of ASW was added to each conditioned jar using a graduated cylinder and larvae (up to 4 h old; n = 10) were introduced. Jars were placed into outdoor water baths and larvae that were active, settled, metamorphosed and dead were recorded at 0, 2, 4, 6, 12, 18, 24, 30, 36 and 42 h.

### Statistical analyses

Assumptions of normality and homoscedasticity were checked graphically (boxplot and residual plots) for each dataset before testing hypotheses, and data transformations applied when assumptions were violated [39]. Datasets not meeting assumptions post-transformations were analysed using permutational methods (PERMANOVA) [40]. A nested ANOVA was used on raw data to distinguish differences in the proportion of coral rubble between islands (fixed), bays (nested, random) and depth (fixed) in Statistica 10. As *C. foliascens* are only found attached to coral rubble, the proportion of substrate occupied by coral rubble across depths were assessed separately within each bay using one-way ANOVAs and Tukey's HSD tests. To evaluate larval release over time, a repeated measures PERMANOVA using the Bray Curtis resemblance matrix (9999 permutations) on raw data was employed, using day and time of day as variables. Differences in larval swimming speeds over time were assessed using a one-factor PERMANOVA (Euclidean resemblance matrix, 9999 permutations).To assess whether vertical position of larvae differed between light cue treatments, repeated measures PERMANOVA using a Euclidean resemblance matrix (9999 permutations) was performed on both “newly released” and 4 h old larvae logged data sets, using exposure to daylight (top or bottom exposure, or light exclusion) and time as factors. Only data from 0 h to 18 h of the experiment was used to avoid any confounding effects of reduced larval swimming after 18 h post-release on phototactic response. Gregarious settlement of larvae to adult conspecific cues was assessed using repeated measures PERMANOVA (Euclidean resemblance matrix, 9999 permutations) with water treatment type (ASW and ai-ASW) and time as variables. Homogeneity of slopes model (HSM), employing the binomial distribution and logit function was used to detect differences in settlement and metamorphosis patterns across larval conspecific densities. Only data from 20 h to 35 h, a period of accelerated larval settlement and metamorphosis, was used, with larval status frequencies as the dependent variable (i.e. dichotomous; settled/metamorphosed and swimming/dead), larval densities as the categorical factor and time as the continuous predictor. The Hosmer-Lemeshow (HL) test was performed to test the fit of data to the model. To evaluate differences in success of settlement and metamorphosis when exposed to biofilm types (intertidal and subtidal) as a settlement cue, the number of settled and metamorphosed larvae was first combined to represent the proportion of larvae that attached successfully to the substrate over time. Subsequently, a repeated measures PERMANOVA (Euclidean resemblance matrix, 9999 permutations) was performed on the raw data with biofilm type and time as variables.

## Results

### Sponge distribution and substrate composition

Benthic surveys covered a depth range between 165 to -609 cm from the tidal datum (FTD) (Table 1). *Carteriospongia foliascens* was only found attached to coral rubble and occurred over a mean depth range of 67 cm (±11) (mean ± 1 S.E.) to 42 cm (±2) FTD at Little Pioneer Bay. The surveyed depth range for north Juno Bay transect 3 was truncated due to a gentler sloping gradient along the transect length, thus hindering detection of lower limit for *C. foliascens*. Mean *C. foliascens* depth distribution at north Juno Bay was between 74 cm (±14, n_transects_  = 3) and 28 cm (±4, n_transects_  = 2) FTD. No sponges were found in subtidal habitats (<0 cm FTD) at either location (see Figures S1 and S2). The maximum total number of sponges was recorded at a depth range of 40 to 49 cm FTD at Little Pioneer Bay (n_sponges_  = 13) and between 30 to 39 cm FTD at north Juno Bay (n_sponges_  = 46) (Figure S3). *C. foliascens* was more abundant at north Juno Bay (0.19 sponge m^−2^ ±0.01; n_transects_  = 2) than at Little Pioneer Bay (0.05 sponge m^−2^ ±0.01; n_transects_  = 3), corresponding to a 282.14% higher frequency of total sponges (Figure S1, S2 and S3).

**Table 1:** *C. foliascens.*

Location	Belt transect number	Sponge upper limit (cm)	Sponge lower limit (cm)	Depth range of survey (cm)
Little Pioneer	1	63	39	101 to -217
Little Pioneer	2	50	41	118 to -185
Little Pioneer	3	86	47	125 to -193
north Juno	1	89	31	165 to -609
north Juno	2	62	24	124 to -198
north Juno	3	73	53	156 to 53

Depth distribution of adult sponges across three belt transects at Little Pioneer Bay and north Juno bay, with the surveyed depth range at each location reflected in the last column, and depths shown in centimetres (cm) representing depth relative to the tidal datum.

**Figure 1:**
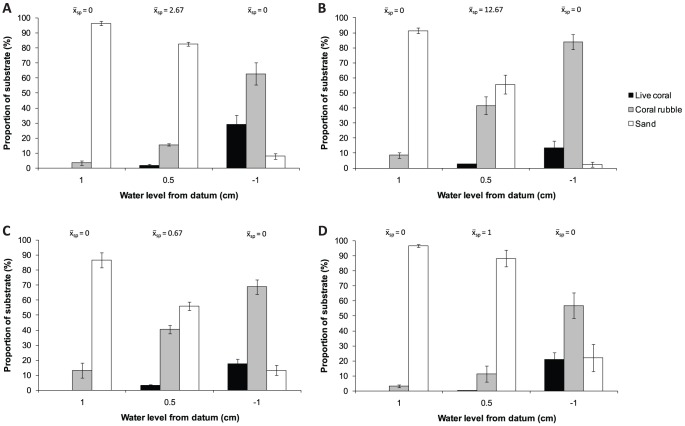
*Carteriospongia foliascens*. Substrate composition (live coral, coral rubble and sand) over three depth profiles (100, 50 and −100 cm relative to tidal datum) at (a) Little Pioneer Bay (Orpheus), (b) Hazard Bay (Orpheus), (c) north Juno Bay (Fantome) and (d) south Juno Bay (Fantome). Mean number of sponges (x-_sp_  =  mean number of individuals/50 m^2^, n_transect_  = 3,) present are highlighted over each respective depth profiles.

A survey of substrate composition was conducted in two bays at both Orpheus and Fantome Island at the mean depth profile where sponges naturally occurred (ca. +50 cm FTD), and at shallow (+100 cm FTD) and subtidal (−100 cm FTD) depth profiles where sponges were absent. Coral rubble composition was lowest at the shallowest depth profile (+100 cm FTD) and increased as water depth increased at all bays (Figure 1). Coral rubble was generally free of macro-invertebrates and macroalgae at both north Juno Bay (proportion bare coral rubble  = 91.0%±0.6) and Little Pioneer Bay (proportion bare coral rubble  = 96.9%±0.6) in January 2012. Live coral was absent in the shallowest depth and increased in cover from +50 cm FTD to −100 cm FTD. Alternatively, sand composition showed a reverse trend occupying a major proportion of the substrate at the shallowest depth and decreasing with increasing depth. The proportion of coral rubble was significantly different between bays (Nested ANOVA: *F*_(2, 30)_  = 15.36, p<0.0001) and depths (Nested ANOVA: *F*_(2, 30)_  = 133.02, p<0.0001), but not between islands (Nested ANOVA: *F*_(1, 30)_  = 117.36, p>0.05). Coral rubble cover in each bay was significantly higher in subtidal depth profiles (−100 cm FTD) compared to other depths (one-factor ANOVAs and Tukey's HSD: p<0.05). The increase in coral rubble cover from shallow to deeper depths provided suitable substrata for *C. foliascens* attachment, however *C. foliascens* was absent in subtidal (+ve FTD) despite this habitat having higher coral rubble cover.

### Larval release and morphological characteristics

**Figure 2:**
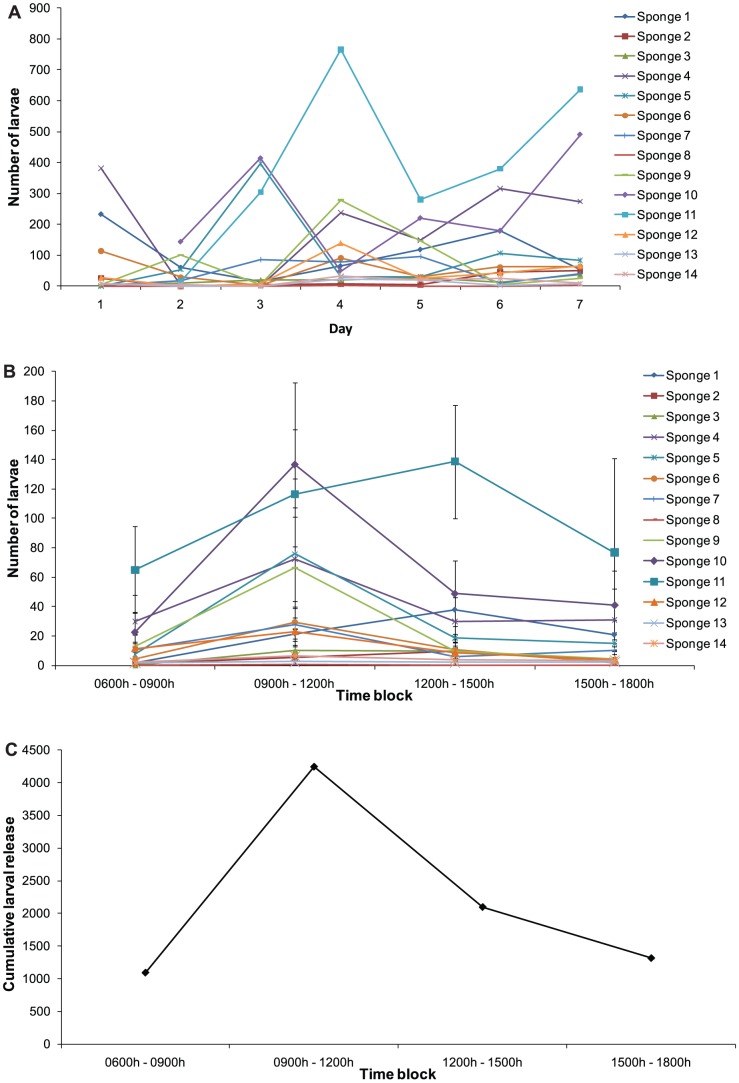
*C. foliascens*. Patterns of larval release showing (a) total daily larval release by individual sponges (n = 14) over 7 days, (b) mean larval release by individual sponges at each time block, and (c) cumulative larval release for all sponges (n = 14) at each time block over 7 days.

*C. foliascens* is viviparous and releases negatively buoyant tufted parenchymella larvae which are cream in colour and possess a dark interior (Figure S4a). Larvae are 850 µm (±3) long, 562 µm (±3) wide and are prolate spheroid in shape (n = 384). Sponge larvae were released between 0600 h and 1800 h, but larval release was variable with a significant interaction between day and time of day (two factor PERMANOVA: pseudo-*F*_(18, 332)_  = 1.746, p<0.001, Figure 2a, b). The intensity of larval release was highest between late morning and noon (0900 h to 1200 h) with sponges releasing up to 122 larvae sponge^−1^ hour^−1^ (Figure 2b). The cumulative frequency of larvae released by all 14 sponges over 7 days was highest between 0900 h and 1200 h (n_larvae released_  = 4241, Figure 2c). Larval release decreased by 25.49% (±5.6) and 31.86% (±6.63) from the peak over early (1200 h to 1500 h; cumulative n_larvae released_  = 2094) and late (1500 h to 1800 h; cumulative n_larvae released_  = 1312) afternoon respectively. Larval release was lowest in the early morning (0600h to 0900h; maximum 60 larvae sponge^−1^ hour^−1^; cumulative n_larvae released_  = 1090). The mean daily larval release was 89 larvae sponge^−1^ day^−1^ (n_sponges_  = 14, n_days_  = 7), with the maximum number of larvae released by an individual being 765 larvae sponge^−1^ day^−1^. No larvae were released during the night. A total of 233 and 887 larvae were estimated to be released per square metre of substrate at Little Pioneer Bay and north Juno Bay respectively over the four most reproductive months (sponge density [sponge m^−2^] * proportion of female sponges [0.43] * mean daily larval release per sponge [larvae sponge^−1^ day^−1^] * days [122 days]; September to December, [38]).

### Larval swimming ability

**Figure 3:**
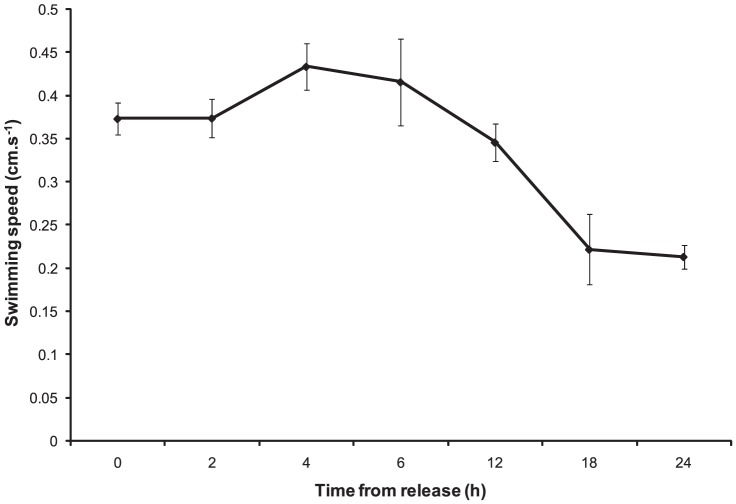
*C. foliascens*. Graph of larval swimming speeds (cm.s^−1^) from release (0 h) to 24 h post-release.

Larval swimming speed at release was 0.37 cm.s^−1^ (±0.02) and fluctuated between 0.35 cm.s^−1^ (±0.02) and 0.43 cm.s^−1^ (±0.03) between 2 h to 12 h post-release (Figure 3). Pair-wise comparisons of swimming speed as larvae age showed a significant decrease in speed after 18 h post-release (0.22 cm.s^−1^ ±0.04) (PERMANOVA: pseudo-*F*_(6, 61)_  = 8.95, p<0.001).

### Pre-settlement behaviour

#### Behaviour of “newly released” larvae

**Figure 4:**
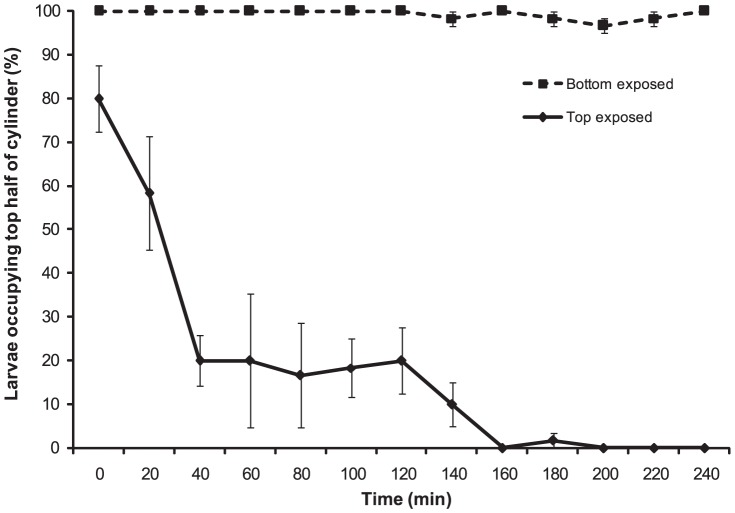
*C. foliascens*. Mean percentages (±1 SE, n = 3, 20 individual replicate^−1^) of larvae (<1 h old at start of experiment) occupying top half of the experimental water column (1000 ml graduated cylinder) when exposed to partial light over 240 minutes. Bottom exposed refers to light presented to the bottom half of the column and top exposed refers to light presented to the top half of the column.

Larvae were negatively phototactic within 40 min of release (Figure 4). When light was presented to the top half of the water column (top exposed; Figure 4), larvae (<1 h old) initiated bottom migration immediately with 80% (±5.77) occupying the darker bottom half of the experimental column at 40 min. Larvae maintained position in the darker region of the column for the rest of the experiment. When light was focussed on the bottom half of the column (bottom exposed; Figure 4) larvae (>96.67%±1.67) maintained their position at the surface over the 240 min duration of the experiment. There was significant interaction of treatment (top/bottom exposure) and time on vertical migration of “newly released” larvae (two factor PERMANOVA: pseudo-*F*_(10, 44)_  = 59.45, p<0.001). This shows that the effect of light conditions on larval positioning in the experimental column is subject to changes over time, with an increasing effect of light conditions on larval positioning as time progressed (Figure 4).

#### Behaviour of 4 h old larvae

**Figure 5:**
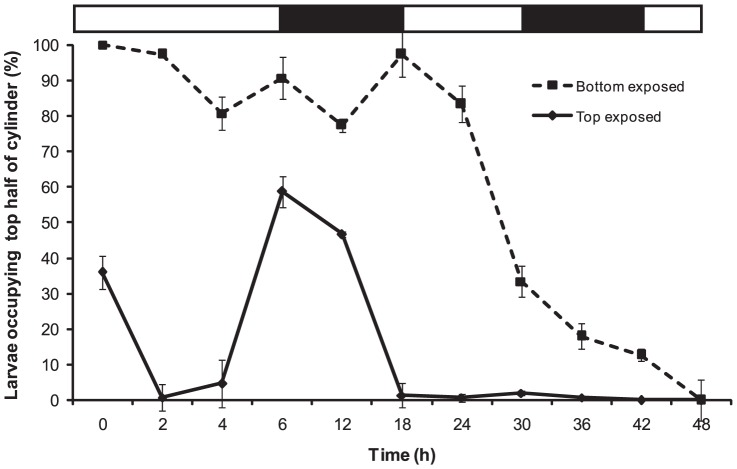
*C. foliascens*. Mean percentages (±1 SE, n = 3, 50 individual replicate^−1^) of larvae (4 h old at start of experiment) occupying top half of the experimental water column (1000 ml graduated cylinder) when exposed to partial light over 48 hours. Bottom exposed refers to light presented to the bottom half of the column and top exposed refers to light presented to the top half of the column. Light and dark bars over the graph represent natural photoperiod, where light is day and dark is night.

*C. foliascens* larvae remained negatively phototactic with age (Figure 5) as demonstrated by a significant interaction of treatment (top/bottom exposure) and time contributing to larval vertical migration (two way PERMANOVA: pseudo-*F*_(5,24)_  = 25.61, p<0.001). When the bottom half of the column was uncovered (bottom exposed), larvae (4 h old; >80.67%±2.40) maintained their position at the surface for the first 24 h of the experiment (28 h post-release) regardless of daylight conditions (day and night represented by white and black horizontal bars; Figure 5). Most larvae (66.67%±2.91) occupied the bottom half of the column at 30 h with all larvae (100%±0) positioned at the bottom of the column at 48 h. When light was focussed on the top half of the column (top exposed; Figure 5), larvae (>95.33%±1.33) immediately migrated to the bottom and maintained this position in the dark for the first 4 h. When light was first excluded from the experiment during the night (6 h), up to 58.67% (±2.91) of larvae migrated towards the exposed top half of the column before returning to the bottom at sunrise (at 18 h of experiment; Figure 5). By 30 h the majority of larvae (>98.67%±0.67) occupied the bottom of the column, and all larvae (100%±0) had migrated to the bottom by the completion of the experiment at 42 h.

**Figure 6:**
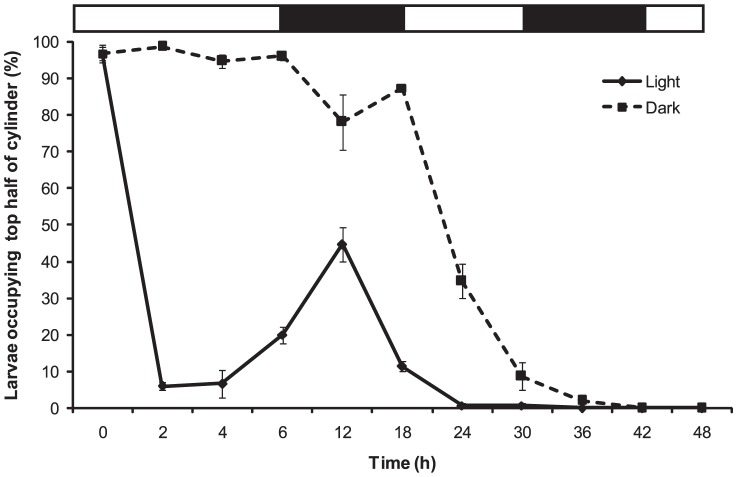
*C. foliascens*. Mean percentages (±1 SE, n = 3, 50 individual replicate^−1^) of larvae (4 h old at start of experiment) occupying top half of the experimental water column (1000 ml graduated cylinder) when exposed to completely dark and light conditions over 48 hours. Light and dark bars over the graph represent natural photoperiod, where light is day and dark is night.

When light was completely excluded from the water column (dark; Figure 6), >78.00% (±7.57) of larvae (4 h old) congregated at the surface for up to 18 h (22 h post-release). Initiation of bottom migration occurred at 24 h when the majority of larvae (65.33%±4.67) moved to the bottom half of the column, with completion of bottom migration achieved at 42 h (100%±0) (Figure 6). Larvae migrated to the bottom immediately in the presence of light (light; Figure 6) where 93.33%±3.71 of larvae occupied the bottom between 2 and 4 h. Similar to the previous experiment, larvae initiated upwards migration during the first nightfall with up to 44.67%±4.67 of larvae found in the top half of the column between 6 h and 18 h (Figure 6). The majority of larvae (88.67%±1.33) returned to the bottom of the column at sunrise (18 h) and maintained this position until bottom migration was completed at 36 h (100%±0). There was a significant interaction of treatment (light/dark) and time between the two treatment groups (two factor PERMANOVA: pseudo-*F*_(5,24)_  = 20.81, p<0.001). This shows that there is a changing effect of light conditions on larval positioning in experimental columns over time, with diminishing effects of light conditions as time progressed (Figure 6).

### Settlement behaviour

#### Gregariousness

**Figure 7:**
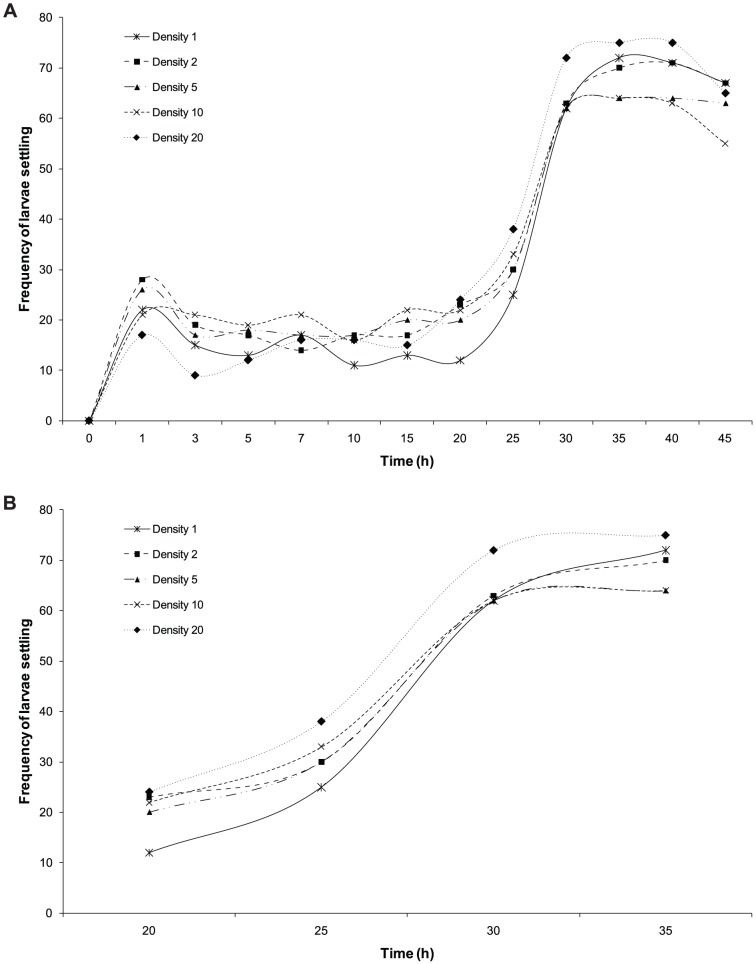
*C. foliascens*. Total frequencies of combined settlement and metamorphosis at larval densities of 1, 2, 5, 10 and 20 larvae well^−1^ over (a) the entire 45 h duration of the experiment and (b) as settlement and metamorphosis accelerated between 20 h and 35 h of the experiment.

Patterns of settlement and metamorphosis (Figure S4b, see Video S1 for a video clip of *C. foliascens* metamorphosis) in *C. foliascens* were similar when larvae were presented with artificial seawater (ASW, control) and adult conspecific seawater (ai-ASW, treatment) (two factor PERMANOVA: pseudo-*F*_(1,160)_  = 0.63, p>0.05). Settlement and metamorphosis occurred as early as 1 h from the start of experiment, where both the control and treatment achieved similar levels of metamorphosis (at 45 h) of 45.56%±5.80 and 42.22%±5.47 respectively. There was no effect of larval densities on settlement and metamorphosis, with all density treatments showing similar settlement and metamorphic trajectories over time (HSM: Wald  = 7.16, df  = 4, p>0.05; HL  = 10.036, p>0.05, Figure 7a, b).

#### Effects of biofilm origin on settlement and metamorphosis

**Figure 8:**
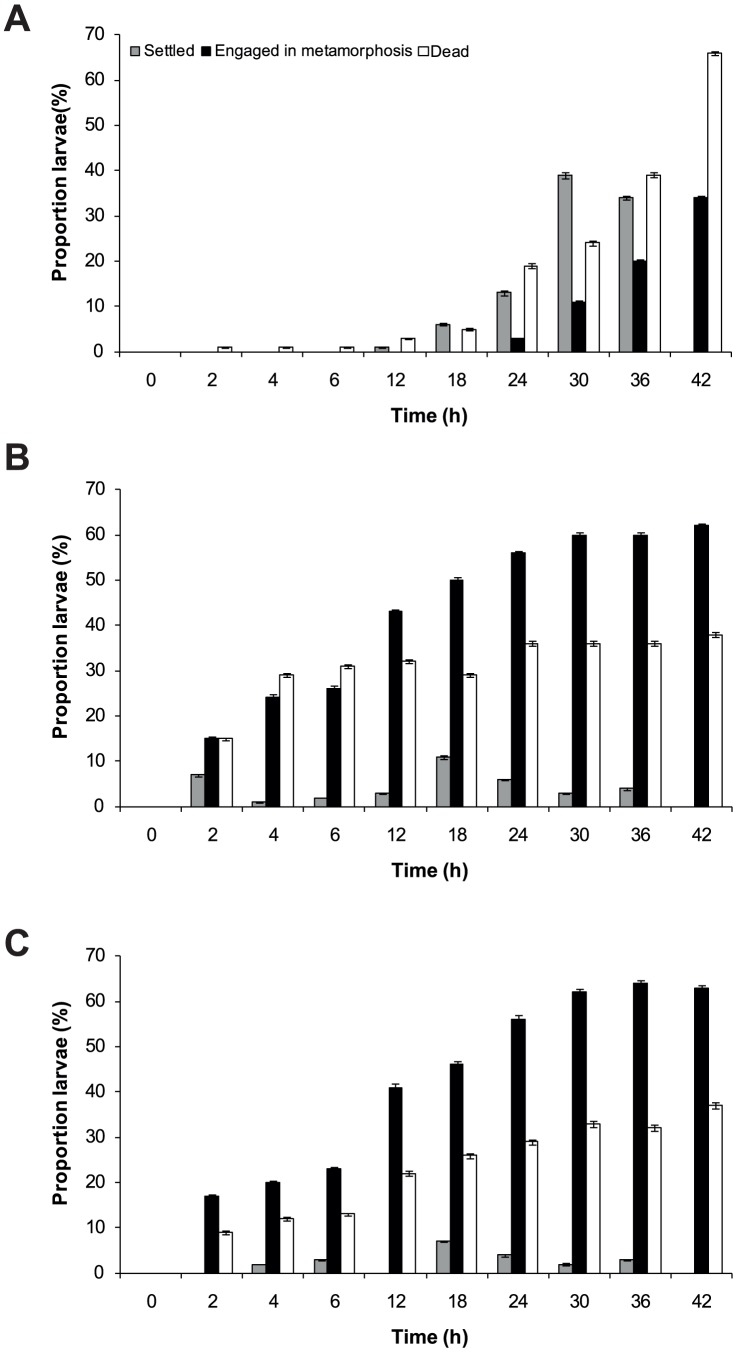
*C. foliascens*. Mean percentages (±1 SE, n = 10 treatment^−1^, 10 individual replicate^−1^) of larvae (4 h old at start of experiment) that were settled, metamorphosed, or dead when presented with a surface that was (a) sterile, (b) conditioned with intertidal biofilm, and (c) conditioned with subtidal biofilm. Surface supporting biofilm were conditioned for duration of six weeks.

Surfaces conditioned with microbial biofilms accelerated settlement and increased metamorphosis in *C. foliascens* when compared to sterile surfaces with a significant interaction of treatment and time (two factor PERMANOVA: pseudo-*F*_(18,270)_  = 3.16, p<0.001). The onset of larval settlement occurred at 12 h when biofilm was absent (Figure 8). Metamorphosis occurred as early as 2 h with 15% (±0.60) and 17% (±0.37) settlement respectively, when intertidal and subtidal biofilms were presented to larvae. Larval metamorphosis at 42 h approached a two-fold increase when biofilms were present compared to sterile surfaces (sterile  = 34%±0.45, intertidal biofilm  = 62%±0.53 and subtidal biofilm  = 63%±0.70).There was no significant effect of the intertidal or subtidal origin of the biofilm on settlement and metamorphosis (pairwise PERMANOVA: t = 0.69, p>0.05).

## Discussion

This study identifies the potential importance of larval behaviour to dispersal, recruitment and adult distributions of intertidal *Carteriospongia foliascens*. Light cues the release of larvae in *C. foliascens*, supporting the importance of light for spawning as demonstrated in other sponges [14]–[16], [41], [42]. The number of larvae released by *C. foliascens* is low when compared to estimates of larval release for the Mediterranean sponges *Corticium candelabrum* (543 to 2063 larvae m^−2^ vs 507,000 larvae m^−2^, [43]) and *Ircinia oros* (765 larvae sponge^−1^ day^−1^ vs 2350 larvae sponge^−1^ day^−1^, [13]). In contrast, *C. foliascens* maximum larval release is similar to those reported for other co-occurring dictyoceratid Great Barrier Reef (GBR) species (i.e. *Luffariella variabilis*, 830 larvae sponge^−1^ day^−1^, [14]; *Rhopaloeides odorabile*, 800 larvae sponge^−1^ day^−1^, [15] and *Coscinoderma matthewsi*, 595 larvae sponge^−1^ day^−1^, [16]). Notably, the *C. foliascens* strategy of continued larval release over several months is unusual compared to those reported for co-occurring GBR sponge species which exhibit spawning periods of several weeks [14]–[16]. The extended spawning period of *C. foliascens* [38], and concomitant higher potential for larval supply, reflects patterns observed for abundant Red Sea sponges species *Niphates* sp. and *Chalinula* sp. [44], [45]. Year-round spawning is likely to be a factor contributing to the abundance of *C. foliascens* on the GBR where they can represent up to 80% of total sponge abundance and biomass [35], [36], [46].

Adult populations of *C. foliascens* can occur from the intertidal to mesophotic zones [35]–[37], [46]. However, the strict depth distribution of adult *C. foliascens* to the intertidal is distinctive of inshore populations (this study). Strict day time larval release, coupled with the early onset of negative phototaxis suggests that larvae move to the benthos within 40 minutes, and may settle within hours from release as shown in other photophobic larval sponge species (e.g. *Xestospongia bocatorensis*, [47]). This behaviour may restrict the capacity of larvae to disperse away from natal habitats, thereby promoting conditions for endogenous recruitment of *C. foliascens*, a phenomenon also seen in scleractinian corals [48], [49]. While larval pre-settlement behaviours, coupled with early competency for settlement, supports limited dispersal potential for this species, contradicting evidence based on laboratory results indicate larvae have the potential to disperse beyond the intertidal habitat of adults.

The ability of larvae to vertically migrate to the surface at the onset of nightfall (up to 60% of larvae, within 8 h from release), suggests that larvae released later in the afternoon, or those that have not settled prior to nightfall, have the potential for dispersal overnight away from intertidal natal habitats and onto adjacent shallow subtidal reefs less than 100 m away. Importantly, daily spawning over several months exposes larvae to variable hydrodynamic conditions (i.e. tide changes, wind-driven surface currents) which can also facilitate larval dispersal over a range of spatial scales [12], [50]. To date, no evidence of geotactic or barotactic mechanisms have been found in sponge larvae. However, the spiralling swimming behaviour of sponge larva, coupled with the differential weighting of larvae through the accumulation of a posterior spicule mass have been proposed to initiate responses similar to geo- or barotaxis in species possessing spicules [51], [52]. Being aspiculate, *C. foliascens* larval migration to the surface upon removal of a light cue, supports alternative mechanisms that initiate geo- or barotactic-like response (e.g. differential weighting of larvae through skeletal development, lipid aggregation or formation of dense proteins), which may interact with larval phototaxis in stimulating directional vertical swimming as seen in the larvae of corals, gastropods and polychaetes [53]–[55]. The eventual bottom migration of negatively buoyant larvae at 30 h post-release reflects the reduction of larval swimming vigour and increased likelihood for settlement at this time. Nevertheless, if larval dispersal is limited, the possibility of a stepping stone model of dispersal at the small geographic scale investigated is highly likely, and should translate to wider horizontal and depth distribution for *C. foliascens* [56], [57]. The use of narrow experimental columns for vertical migration and phototaxis studies, exposes larvae to static water conditions that are unrepresentative of natural hydrodynamics, and may introduce wall effects influencing larval behaviour. However, these effects can be minimized [58]–[60].

This study shows that despite limited dispersal potential, intertidal *C. foliascens* larvae should be able to disperse to neighbouring subtidal habitats. The abundance of suitable and available settlement substrate in the subtidal (i.e. bare coral rubble), coupled with the capacity of *C. foliascens* larvae to successfully metamorphose to subtidal biofilms indicates subtidal habitats are potential recruitment environments. The absence of adult *C. foliascens* on inshore subtidal reefs of the Great Barrier Reef (GBR) potentially reflects the role that post-settlement processes have on *C. foliascens* population distributions.

*C. foliascens* possess symbiotic cyanobacteria, and can rely on phototrophic processes to supply more that 50% of their nutrition [34], [61]. The influence of light limitation on depth distribution of phototrophic sponges, including *C. foliascens*, have been reported for populations at Davies Reef (120 km south-east offshore from the study site), with the highest abundance between 10 and 30 m, and significant reductions outside of this range [62]. While reduced light penetration demarcated distributions with depth, lower numbers of sponges in shallow water was indicative of a physical disturbance (i.e. wave exposure and turbulence) that can detach sponge species having a single point of substrate attachment (e.g. stalk in *C. foliascens*) [62].

Unlike offshore locations like Davies Reef, inshore reefs are subjected to high terrestrial run-off which can contribute to elevated levels of suspended particles (i.e. fine clay and organic particles), leading to mortalities of sponge recruits through the smothering of the aquiferous system [63], and reducing light penetration to the subtidal region [64]. In addition, associated eutrophication also accelerates phytoplankton production contributing to increased turbidity [64], [65]. Water quality, particularly increased turbidity at inshore environments is likely to impede photosynthesis and subsequently successful recruitment of *C. foliascens* to deeper subtidal reefs, a process reported previously in other benthic photoautotrophs such as scleractinian corals [66], [67]. While the colonization of *C. foliascens* to the subtidal may be restricted by lowered light penetration, abundance of *C. foliascens* in the intertidal at the study site may be supported by habitat characteristics (within bays) which shelter sponges from negative impacts of turbulent hydrodynamics [62]. In addition, other post-settlement processes such as predation [26], [27], [68]–[70] and space competition [71], [72] may also contribute to the absence of adult *C. foliascens* in the subtidal. While population genetic data and transplantation experiments would add considerably to our understanding of connectivity in *C. foliascens* populations across horizontal (i.e. inshore and offshore) and vertical distances (i.e. depths), evidence from this study suggests self-recruitment of individuals to the intertidal at inshore reefs for this species [2], [50].

## Supporting Information

Figure S1 ***Carteriospongia foliascens*** **.** Depth profile (line, cm) from the tidal datum (FTD) along a 500 m transect, and corresponding sponge abundance (grey bars, frequency) at every 10 m distance from the edge of mangroves at Little Pioneer Bay. a, b and c represents transects 1, 2 and 3 respectively.
(EPS)

Figure S2 ***C. foliascens*** **.** Depth profile (line, cm) from the tidal datum (FTD) along a 500 m transect, and corresponding sponge abundance (grey bars, frequency) at every 10 m distance from the edge of mangroves at north Juno Bay. a, b and c represents transects 1, 2 and 3 respectively.
(EPS)

Figure S3 ***C. foliascens*** **.** Sponge abundance (total number) at specific depth ranges for Little Pioneer Bay and north Juno Bay. Depth ranges excluded from this graph are those not supporting any sponges.
(EPS)

Figure S4 ***C. foliascens*** **.** Micro-photographs showing (a) a typical larva with pigmented posterior ring (pr) and dark interior (di), and (b) a settled larvae with anterior end attached to the experimental substrate and undergoing metamorphosis, invagination occurs at the pigmented posterior ring (pr) and disappears when larva is engaged in metamorphosis, forming a distinct flattening of the posterior half to assume a dome-like morphology (see Video S1 for a complete time-lapse video of *C. foliascens* engaging in metamorphosis).
(EPS)

Video S1 **Time-lapse video of** ***C. foliascens*** **larva engaging in metamorphosis.**
(AVI)
